# Toward Strong Near‐Infrared Absorption/Emission from Carbon Dots in Aqueous Media through Solvothermal Fusion of Large Conjugated Perylene Derivatives with Post‐Surface Engineering

**DOI:** 10.1002/advs.202202283

**Published:** 2022-06-02

**Authors:** Yupeng Liu, Josh Haipeng Lei, Gang Wang, Zhiming Zhang, Jun Wu, Bohan Zhang, Huiqi Zhang, Enshan Liu, Liming Wang, Tzu‐Ming Liu, Guichuan Xing, Defang Ouyang, Chu‐Xia Deng, Zikang Tang, Songnan Qu

**Affiliations:** ^1^ Joint Key Laboratory of the Ministry of Education Institute of Applied Physics and Materials Engineering (IAPME) University of Macau Taipa Macau SAR 999078 China; ^2^ Cancer Center Faculty of Health Sciences University of Macau Taipa Macau SAR 999078 China; ^3^ MOE Frontier Science Centre for Precision Oncology University of Macau Taipa Macau SAR 999078 China; ^4^ State Key Laboratory of Quality Research in Chinese Medicine and Institute of Chinese Medical Sciences University of Macau Taipa Macau SAR 999078 China

**Keywords:** carbon dots, near‐infrared absorption, near‐infrared bioimaging, near‐infrared emission, perylene derivatives, two‐photon fluorescence

## Abstract

Carbon dots (CDs) have attracted significant interest as one of the most emerging photoluminescence (PL) nanomaterials. However, the realization of CDs with dominant near‐infrared (NIR) absorption/emission peaks in aqueous solution remains a great challenge. Herein, CDs with both main NIR absorption bands at 720 nm and NIR emission bands at 745 nm in an aqueous solution are fabricated for the first time by fusing large conjugated perylene derivatives under solvothermal treatment. With post‐surface engineering, the polyethyleneimine modified CDs (PEI‐CDs) exhibit enhanced PL quantum yields (PLQY) up to 8.3% and 18.8% in bovine serum albumin aqueous and DMF solutions, which is the highest PLQY of CDs in NIR region under NIR excitation. Density functional theory calculations support the strategy of fusing large conjugated perylene derivatives to achieve NIR emissions from CDs. Compared to the commercial NIR dye Indocyanine green, PEI‐CDs exhibit excellent photostability and much lower cost. Furthermore, the obtained PEI‐CDs illustrate the advantages of remarkable two‐photon NIR angiography and in vivo NIR fluorescence bioimaging. This work demonstrates a promising strategy of fusing large conjugated molecules for preparing CDs with strong NIR absorption/emission to promote their bioimaging applications.

## Graphical abstract

Carbon dots with both main near‐infrared (NIR) absorption and emission bands in aqueous solution are fabricated through solvothermal fusion of large conjugated perylene derivatives. After post‐surface modification with polyethyleneimine, high photoluminescence quantum yields in NIR region up to 8.3% and 18.8% are obtained in their bovine serum albumin aqueous and DMF solutions, respectively.

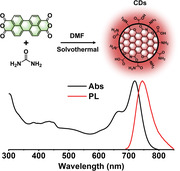

## 1 Introduction

Carbon dots (CDs) are a special type of 0D luminescent carbon nanomaterial with sizes of less than 10 nm^[^ ^1^ ^]^ and optical properties that can be modulated by the sp^2^ conjugated domains,^[^ ^2^ ^]^ doping elements,^[^ ^3^ ^]^ and surface chemical structures.^[^ ^4^ ^]^ Because of their good biocompatibility, low toxicity, good photostability, and low cost, CDs have distinct advantages in fluorescent bioimaging.^[^ ^5^ ^]^ Compared with the visible light region (400–700 nm), the near‐infrared (NIR) window (700–1700 nm) has a distinct advantage in high‐contrast in vivo fluorescence imaging owing to its deeper tissue penetration, lower tissue self‐absorption/scattering, and less autofluorescence.^[^ ^6^ ^]^ To date, various CDs have been developed and efficient blue, green, and red emissions have been achieved in several CDs systems.^[^ ^7^ ^]^ However, efficient NIR‐emissive CDs have rarely been reported.^[^ ^8^ ^]^ The initial attempt was to synthesize NIR‐emissive CDs from high price NIR‐emissive organic dyes.^[^ ^9^ ^]^ However, the PLQYs of the reported NIR‐emissive dye‐derived CDs are still less than 10% in aqueous solutions. For example, Jing and colleagues^[^ ^9a^ ^]^ used hydrophobic cyanine dyes (CyOH), a commercial NIR fluorescent dye, as a precursor to prepare NIR‐emissive CDs. The prepared CDs demonstrated the optical properties of CyOH, while the PLQY in the NIR region was only 5.7% in organic solvents, which may be even lower in aqueous solutions.

To achieve efficient NIR emission, tuning the main absorption band of CDs to the NIR region is necessary.^[^ ^8^, ^10^ ^]^ Significant efforts have been made to regulate the bandgap of CDs to longer wavelengths by increasing the size of the conjugation domain in the carbon cores or by introducing electron‐withdrawing groups through surface engineering.^[^ ^11^ ^]^ In our previous work,^[^ ^11a,b^ ^]^ the main absorption of the CDs was red‐shifted to 724 nm by microwave‐assisted exfoliation and the introduction of electron‐withdrawing environments. An emerging NIR emission band at 784 nm was observed with a PLQY of 11% in DMF, while their NIR absorption and emission bands disappeared in aqueous solution. In these surface electron‐withdrawing group‐treated CD systems, efficient NIR absorption/emissions were only achieved in organic solvents, such as ethanol, DMF, and DMSO, but significantly vanished in aqueous solution, which greatly hinders in vivo NIR fluorescent imaging applications. To the best of our knowledge, there are no reports of CDs with both main absorption and emission bands in the NIR region in aqueous solutions.

Based on theoretical calculations, increasing the diameter of the conjugated sp^2^ domain to ≈2 nm could tune its bandgap to the NIR region.^[^ ^12^ ^]^ Reported CDs are usually synthesized from non‐ or low‐conjugated molecules through dehydration and carbonization processes.^[^ ^13^ ^]^ Although their particle sizes can be much larger than 2 nm, their main absorption bands are always observed in the UV or visible regions, indicating smaller conjugated sp^2^ domains in the carbon cores.^[^ ^14^ ^]^ Thus, carbon cores with large conjugation are thought to be the primary factor for CDs with both main NIR absorption/emission in aqueous solution.

Perylene derivatives, a kind of cheap organic dyes containing five conjugated aromatic rings, are potential candidates to construct CDs with large conjugated carbon cores. However, due to their low solubility in dimer or polymer, the synthesis of perylene‐based CDs has not been reported. In this work, we unprecedentedly proposed a strategy of synthesis of strong NIR emissive CDs through fusing large conjugated perylene derivative of 3,4,9,10‐perylenetetracarboxylic dianhydride (PTCDA) to construct large conjugated carbon cores with a narrowed bandgap in NIR region. With the help of urea, PTCDA can fuse together in DMF under solvothermal conditions to give rise to a new type of perylene‐derived CDs, which exhibit unprecedented both main NIR absorption and emission peaks in aqueous solution. After modification with polyethyleneimine (PEI) through post‐solvothermal treatment in DMF, the PEI‐CDs exhibited enhanced PLQYs in the NIR region of up to 18.8% and 8.3%, which were demonstrated in their DMF and bovine serum albumin (BSA) aqueous solutions, respectively. To the best of our knowledge, this is the highest reported PLQY in the NIR region under NIR excitation from CDs in aqueous media. Density functional theory (DFT) calculations further support the strategy of fusing large conjugated perylene derivatives to achieve narrowed bandgap NIR emission from CDs with expanded conjugation. The prepared PEI‐CDs exhibit low cellar toxicity and quick renal clearance, which can be visualized in vivo and in vitro main organs NIR fluorescent imaging, demonstrating the high tissue penetration of the NIR ex/emissions from PEI‐CDs. Furthermore, the as‐prepared PEI‐CDs with low‐cost exhibit excellent photostability and two‐photon NIR fluorescence properties. Under NIR II fs laser (1300 nm) excitation, two‐photon NIR fluorescence angiography of mouse ears has been realized via intravenous injection of PEI‐CDs aqueous solutions.

## 2 Results and Discussion

CDs were synthesized by reacting PTCDA (0.2 g) and urea (1 g) at 160 °C for 5 h under solvothermal conditions in 20 mL DMF and then cooled to room temperature. The obtained upper layer of the dark brown solution was diluted and then purified using a dialysis bag of 1000 Da for 24 h to remove residual organic molecules. The aqueous solution in the dialysis bag was collected and freeze‐dried to obtain the dark product of the CDs. PEI‐modified CDs (PEI‐CDs were prepared by post‐solvothermal treatment of the first reacted up layer raw solution with 0.5 g PEI at 160 °C for 5 h. The obtained dark brown solution was diluted and then purified in a dialysis bag of 20 kDa for 24 h to remove residual PEI and small organic molecules. The aqueous solution in the dialysis bag was collected and freeze‐dried to obtain PEI‐CDs.

**Figure 1:**
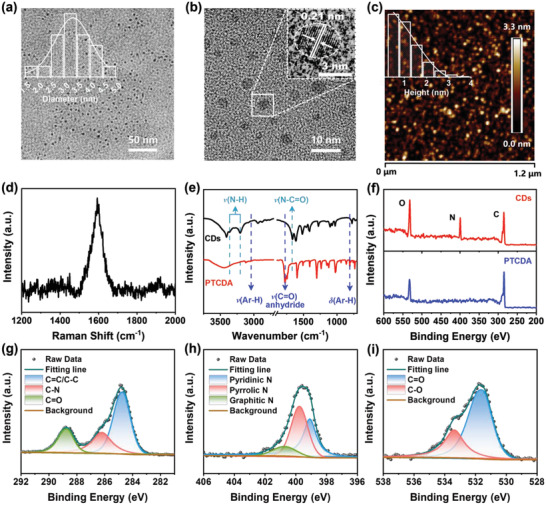
Characterization of CDs. a) TEM images of the CDs with inserted size distribution diagram. b) TEM and HRTEM (insert) images of the CDs. c) AFM image of the CDs and the inset shows the height profile analysis. d) Raman and e) FT‐IR spectra of the CDs. f) XPS survey spectra of the CDs and PTCDA, high‐resolution g) C 1s, h) O 1s, and i) N 1s XPS spectra of the CDs.

Transmission electron microscopy (TEM) and atomic force microscopy (AFM) were used to characterize the morphology of the CDs. The TEM images (**Figure** **1**a,b) illustrated that the prepared CDs were evenly monodispersed with a homogeneous diameter of 3.3 ± 0.7 nm. The high‐resolution TEM (HRTEM) image (Figure 1b, inset) shows clear lattice fringes (0.21 nm), which correspond to the (100) crystal plane of graphene. Moreover, the AFM image reveals the morphologic heights of the CDs in the range of 0.5 to 3.5 nm with an average height of 1.0 nm (Figure 1c), indicating that the CDs primarily consisted of one or a few layers of graphene‐like plates. In the Raman spectra of the CDs (Figure 1d), a sharp peak around 1593 cm^−1^ is observed, which can be ascribed to the in‐plane vibration (G band) of C atoms, while no clear disordered “D” band appears around 1380 cm^−1^, indicating the formation of ordered graphene‐like carbon cores.^[^ ^15^ ^]^

The chemical structures of the CDs were further identified by Fourier transform infrared (FT‐IR) spectrometer and X‐ray photoelectron spectroscopy (XPS). A comparison of the FT‐IR spectra of the CDs and precursor PTCDA (Figure 1e) shows new absorption bands at ≈3365 and 3205 cm^−1^, which correspond to the stretching vibration of N—H. Additionally, the peaks at ≈3060 and 810 cm^−1^ were assigned to the stretching and bending vibrations of Ar—H, respectively. The descent of these two peaks demonstrated the fusion of PTCDA during the solvothermal reaction. The peak of the stretching vibration of C═O at around 1775 cm^−1^ in anhydride^[^ ^16^ ^]^ vanishes, while the stretching vibration of O═C—N at around 1664 cm^−1^ arises,^[^ ^17^ ^]^ indicating a reaction between PTCDA and urea, which could increase the solubility of the fused conjugated carbon cores.

In the XPS spectra of the CDs, three peaks were attributed to C (285.18 eV), N (399.54 eV), and O (531.60 eV) (Figure 1f). Compared with PTCDA, the appearance of N and increased O content indicates that urea participated in the formation of the CDs (Figure 1f). The high‐resolution XPS C1s peak of the CDs (Figure 1g) could be deconvoluted into three peaks: C—C/C═C (≈284.80 eV), C—N (≈286.29 eV), and C═O (≈288.71 eV).^[^ ^18^ ^]^ The N 1s of the CDs in Figure 1e exhibits three peaks ascribed to pyridinic N (≈399.05 eV), pyrrolic N (≈399.78 eV), and graphitic N (≈400.78 eV), suggesting that N was doped into the graphene cores.^[^ ^19^ ^]^ The O1s spectra of the CDs (Figure 1f) showed two peaks at ≈531.58 and ≈533.39 eV which could be attributed to C═O and C—O, respectively.^[^ ^20^ ^]^

**Figure 2:**
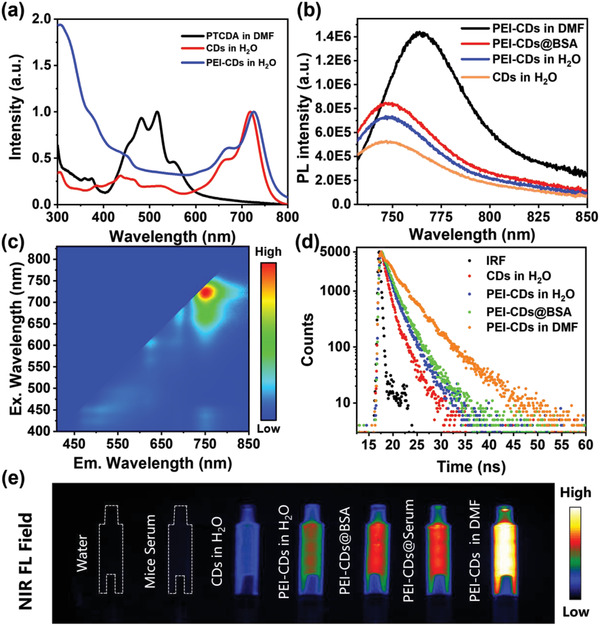
Optical properties of CDs and PEI‐CDs. a) UV–vis absorption spectra of PTCDA in dilute DMF solution and CDs, PEI‐CDs in dilute aqueous solutions. b) PL spectra of CDs, PEI‐CDs PEI‐CDs@BSA in aqueous solutions, and PEI‐CDs in DMF solution under 725 nm excitation. c) Excitation‐emission mapping of PEI‐CDs in dilute aqueous solution. d) PL decay curves of CDs, PEI‐CDs PEI‐CDs@BSA in aqueous solutions, and PEI‐CDs in DMF solution under 640 nm excitation and monitored at 751 nm (IRF = instrument response function). e) NIR fluorescence images of water, mice serum, CDs in H_2_O, PEI‐CDs in H_2_O, PEI‐CDs@BSA, PEI‐CDs@Serum, and PEI‐CDs in DMF (from left to right) under 690 nm excitation and the emission was recorded with a 750 nm long‐pass optical filter.

PTCDA only shows the main absorption bands in the visible range, peaking at 452, 481, and 516 nm in the DMF solution. The CDs interestingly exhibit main NIR absorption bands centered at 720 nm in aqueous solutions, indicating extended conjugated sp^2^ domains in the carbon cores compared to their precursors (**Figure** **2**a). Notably, without urea, a single precursor of PTCDA cannot form CDs with the main NIR absorption band under the same solvothermal treatment (Figure S5a, Supporting Information), nor do the reaction between PTCDA and urea under hydrothermal conditions (Figure S5b, Supporting Information). A fusing process occurred between PTCDA and urea under solvothermal conditions, leading to the formation of CDs with larger *π*‐conjugation. After surface modification with PEI, the absorption bands in the NIR region of the as‐prepared PEI‐CDs were slightly red‐shifted to 726 nm (Figure 2a), whereas the absorption bands in the UV region were obviously enhanced, which can be attributed to the surface‐modified PEI chains.

PTCDA did not exhibit NIR emission, whereas the prepared CDs exhibited clear excitation independent NIR emission centered at 745 nm in the aqueous solution (Figure S6a, Supporting Information) with a PLQY of 3.3% under 720 nm excitation (**Table** **1**). After modification with PEI, the NIR emission from PEI‐CDs was enhanced with a PLQY of 5.3% in aqueous solution under the excitation of 725 nm, while a much higher PLQY of up to 18.8% was observed in the DMF solution under the excitation of 741 nm. The excitation‐emission mapping of PEI‐CDs in aqueous solution exhibited an excitation‐independent NIR emission center at 751 nm, which was red‐shifted to 765 nm in DMF (Figure 2c; Figure S6c, Supporting Information). Considering the improved PLQY after modification with PEI in aqueous solution and the much higher PLQY in their aprotic organic solution, the NIR emission from the CDs in aqueous media can be efficiently enhanced by preventing the interactions of water molecules with their conjugated carbon cores. Thus, BSA, a widely used biocompatible protein, was selected for combination with PEI‐CDs.^[^ ^21^ ^]^ After adding BSA, the NIR emissions from PEI‐CDs were significantly enhanced, indicating that BAS molecules could be absorbed on the surface of PEI‐CDs to further prevent fluorescence quenching by water molecules. It is exciting to find that PLQY up to 8.3% in the NIR region can be achieved in PEI‐CDs BSA (PEI‐CDs@BSA) aqueous solution (PEI‐CDs: 500 µg mL^−1^, BSA: 15 mg mL^−1^), which is the highest PLQY of CDs in NIR region in aqueous media (Table S1, Supporting Information).

**Table 1:** Emission peaks, PLQYs, and lifetimes of the CDs, PEI‐CDs, PEI‐CDs@BSA in dilute aqueous solutions, and PEI‐CDs in DMF

	Solvent	*λ*_Abs/_ *λ*_Em_ [nm]	PLQY/*λ*_Ex_ [%]/[nm]	*τ*_1_/*A*_1_ [ns]/[%]	*τ*_2_/*A*_2_ [ns]/[%]	*τ*_avg_ [ns]
CDs	H_2_O	720/745	3.3/720	0.95/88.16	3.60/11.84	1.26
PEI‐CDs	H_2_O	726/751	5.3/725	1.30/78.22	3.50/21.78	1.78
PEI‐CDs@BSA	H_2_O	726/750	8.3/725	1.45/69.63	3.50/30.37	2.08
PEI‐CDs	DMF	741/765	18.8/741	2.61/61.56	5.66/38.44	3.78

The PL decay curves for the NIR emissions from the CDs, PEI‐CDs, and PEI‐CDs@BSA at 751 nm under 640 nm excitation are displayed in Figure 2d. The average PL lifetimes of CDs, PEI‐CDs, and PEI‐CDs@BSA in aqueous solutions, as well as the PEI‐CDs in the DMF solution, were measured to be 1.28, 1.78, 2.08, and 3.78 ns, respectively, as shown in Table 1. The gradually increased PL lifetimes after modification with PEI and further combination with BSA indicate effective inhibition of energy dissipation by water molecules, which agrees well with their PL properties. Similarly enhanced NIR emissions from PEI‐CDs were observed in their serum solutions, as shown in Figure 2e.

The photostability of PEI‐CDs was further investigated by comparing them with ICG, a widely used commercial NIR fluorescence (FL) probe. Under 730 nm laser irradiation at 30 nW cm^−2^, both absorption and PL intensity of ICG decayed to 50% within 15 min, while those of PEI‐CDs maintained over 85% (Figure S7, Supporting Information). After 1 h laser irradiation, the emission intensity of ICG dropped sharply to 4%, while the emission intensity of PEI‐CDs was still maintained at 55.2%, indicating much better photostability of PEI‐CDs than ICG. In addition, the synthesis cost of PEI‐CDs is much lower (≈1%) than the price of ICG (Table S2, Supporting Information). The above results indicate that the PEI‐CDs are promising NIR FL probes for in vivo bioimaging applications.

**Figure 3:**
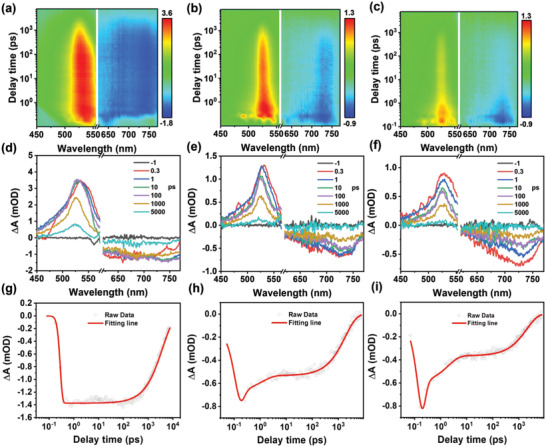
Transient absorption of PEI‐CDs. 2D pseudo‐color maps of the TA spectra of a) PEI‐CDs (50 mg mL^−1^) in DMF, b) PEI‐CDs@BSA (PEI‐CDs: 50 mg mL^−1^, BSA: 50 mg mL^−1^), and c) PEI‐CDs (50 mg mL^−1^) in water with a pump wavelength of 600 nm (1 kHz, 100 fs). TA spectra of d) PEI‐CDs in DMF, e) PEI‐CDs@BSA, and f) PEI‐CDs in water at indicated delay times. Bleach signal kinetics of g) PEI‐CDs in DMF, h) PEI‐CDs@BSA, and i) PEI‐CDs in water for *λ*_pump_ = 600 nm and probe wavelengths of 735 nm.

Femtosecond transient absorption (TA) spectroscopy measurements were also performed to investigate the excited‐state dynamics of PEI‐CDs in DMF and in water with and without BSA. The representative pseudo‐color TA spectra are shown in **Figure** **3**a,d,g) with delay times from −1 to 7600 ps. The negative signals (blue) at 630–760 nm correspond to the ground state bleaching (GSB), which is in general agreement with their steady‐state absorption spectra. The positive signals (red) at 510–550 nm are ascribed to the excited‐state absorption, which exhibits similar decay lifetimes to their corresponding GSB signals. Comparing the TA kinetic traces of their GSB signals at 735 nm, PEI‐CDs in water show a fast decay within 136.9 ps, while PEI‐CDs in BSA aqueous solution and DMF show a much longer lifetime of up to 838.4 and 3862.7 ps, respectively. The fast decay of PEI‐CDs in water can be attributed to the rapid electron‐transfer quenching of the excited state by water molecules. After combining with BSA, PEI‐CDs@BSA in water exhibited a much longer lifetime than PEI‐CDs in water, further demonstrating that the combination with BSA can effectively avoid the energy losses of PEI‐CDs in aqueous solutions by preventing interactions between the conjugated carbon cores and water molecules.

**Figure 4:**
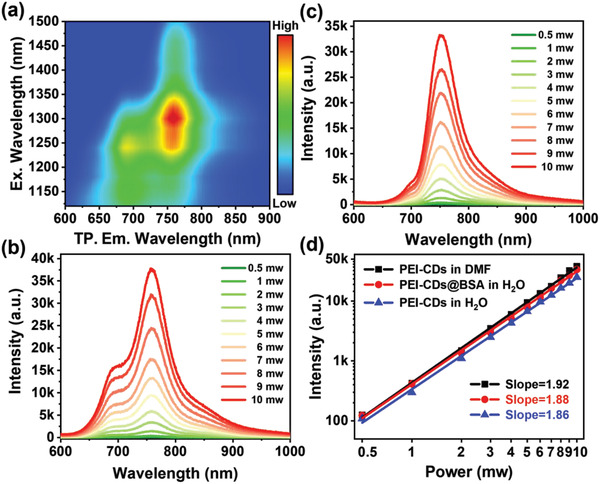
Two‐photon properties of PEI‐CDs. a) Two‐photon excitation‐emission mapping of the PEI‐CDs (50 mg mL^−1^) in DMF solution. Two‐photon emission spectra of the b) PEI‐CDs (50 mg mL^−1^) in DMF solution and c) PEI‐CDs@BSA in aqueous solution, (PEI‐CDs 50 mg mL^−1^, BSA 50 mg mL^−1^). d) The linear relationship between the Briggsian logarithm of the maximum peak intensity and the Briggsian logarithm of different excitation laser powers of PEI‐CDs in DMF, PEI‐CDs@BSA, and PEI‐CDs in aqueous solution, the slope of which are respectively 1.92, 1.88, and 1.86, and are all close to 2.

To gain insight into the two‐photon FL properties of PEI‐CDs, their two‐photon excitation‐emission spectra were investigated. Two‐photon excitation‐emission mapping of the PEI‐CDs in DMF solution (**Figure** **4**a) revealed that the maximum two‐photon NIR emission intensity appeared under 1300 nm fs‐laser excitation. Therefore, a 1300 nm fs pulse laser was employed for the following two‐photo fluorescence spectra and two‐photon NIR imaging investigations. The two‐photon fluorescence spectra of PEI‐CDs in aqueous solutions with and without BSA and PEI‐CDs in DMF solution are displayed in Figure 4c, Figure S8a, Supporting Information, and Figure 4b, respectively. The NIR emission intensities (plotted on logarithmic scales) of PEI‐CDs almost increased linearly with the logarithmic laser power, with slopes close to 2, indicating two‐photon FL behavior (Figure 4d). The two‐photon FL of PEI‐CDs@BSA aqueous solution increased faster than that of pure PEI‐CD aqueous solution but slower than that of PEI‐CDs in DMF solution upon increasing the laser power. These results indicate that the combination with BSA played an important role in preventing FL quenching by water molecules to realize both strong single‐ and two‐photon NIR FL from PEI‐CDs in aqueous solutions.

**Figure 5:**
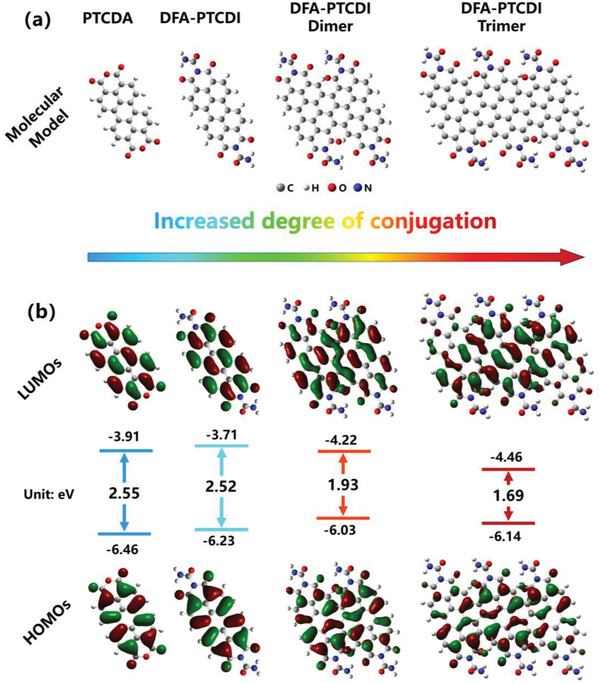
DFT calculations of the four molecular models with increasing conjugation. a) Molecular structures of the four models and b) calculated LUMOs and HOMOs of PTCDA, DFA‐PTCDA, DFA‐PTCDA dimer, and DFA‐PTCDA trimer (from left to right).

To reveal the luminescence mechanism of the NIR‐emissive CDs, we simulated four molecular structures using DFT calculations: PTCDA, urea‐reacted PTCDA (DFA‐PTCDA), the dimer of DFA‐PTCDA, and the trimer of DFA‐PTCDA, as shown in **Figure** **5**a. Geometric model optimization and bandgap calculations of the four molecular structures were performed using the hybrid exchange‐correlation density functional (B3LYP) and 6‐31G(d) basis set in the DFT calculations. The geometrically optimized structures as well as the lowest unoccupied molecular orbital (LUMO) and highest occupied molecular orbital (HOMO) distributions of the four molecular structures in vacuum are shown in Figure 5b. The calculated bandgaps of the four molecular structures were 2.55, 2.52, 1.93, and 1.69 eV, respectively. The reaction with urea cannot change the bandgap of PTCDA, whereas the fusion of the conjugated segments of PTCDA can gradually shift their bandgaps from the green light region to the NIR region. The calculated bandgap of the trimer of DFA‐PTCDI is 1.69 eV, which agrees well with the observed NIR emission center of CDs (1.65 eV) in aqueous solution.

**Scheme 1:**
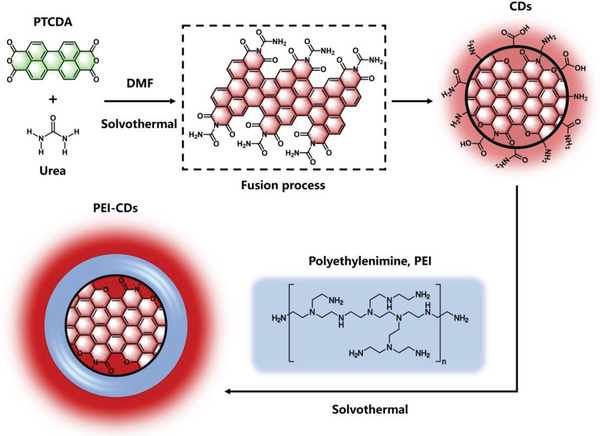
A possible formation process of the CDs and PEI‐CDs.

Based on the above results, a possible formation process from PTCDA to NIR‐emissive CDs and PEI‐CDs is proposed in **Scheme** **1**. Under solvothermal conditions, the conjugated segments of PTCDA covalently fused to form rigid graphene plates with extended *π*‐conjugation, which contributed to the NIR emission from the CDs. After post‐solvothermal treatment by adding PEI, the PEI molecules were modified on the surface of the CDs, which prevented the interactions of water molecules with their conjugated carbon cores, leading to the improved NIR emission in aqueous solutions.

To apply in bioapplications, we investigate their stability in response to some biological parameters such as pH, temperature, ionic species, and ionic strength, the results are shown in Figure S9, Supporting Information. In the pH values from 5 to 12, the fluorescence of PEI‐CDs at 751 nm increased with the increase of pH value, indicating that an acid environment can quench the fluorescence while an alkaline environment can enhance their fluorescence. In the temperature range of 5–95 ℃, the fluorescence intensity of PEI‐CDs was nearly unaffected, during which the NIR fluorescence was slightly enhanced at the body temperature (35–40 ℃). We also explored the NIR fluorescence changes of PEI‐CDs aqueous solutions in the presence of different anions and cations. As shown in Figure S9c, Supporting Information, the fluorescence intensity of PEI‐CDs at 751 nm is less affected in the presence of Li^+^, Na^+^, K^+^, Ca^2+^, Mg^2+^, F^−^, Cl^−^, and Br^−^ (100 µm). The fluorescence intensity of PEI‐CDs decreased slightly with the increasing ionic strength, which may be due to the dissociation of PEI chains on the surface of PEI‐CDs under the larger ionic strength. Even at a high ionic strength of 0.5 mol L^−1^, the fluorescence of PEI‐CDs remained above 60%. The above results indicate that PEI‐CDs have good stability and are suitable for biological applications.

**Figure 6:**
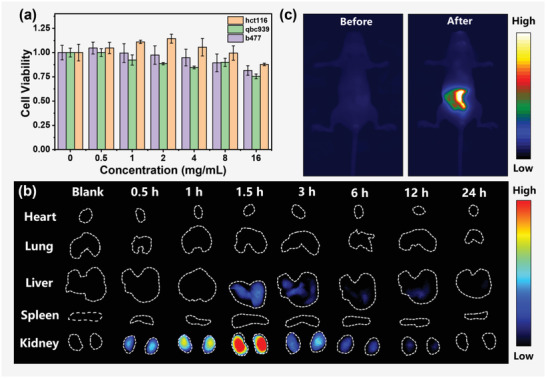
Biocompatibility and in vivo NIR bioimaging. a) Cytotoxicity assessment of PEI‐CDs. b) NIR FL imaging of the major mice organs at several time points before and after the intravenous injections of 200 µL PEI‐CDs aqueous solutions at 15 mg mL^−1^. c) In vivo NIR fluorescence images of a mouse before and after 1 h gavage injection of PEI‐CDs aqueous solution (400 µL, 15 mg mL^−1^). The images were recorded with a 750 nm long‐pass optical filter under 690 nm excitation.

To investigate its biocompatibility, the cytotoxicity of the PEI‐CDs was investigated using hct116, qbc939, and b477 cells. PEI‐CDs did not inhibit cell viability at concentrations up to 16 mg mL^−1^ (**Figure** **6**a); thus, they exhibited low cytotoxicity. Based on the NIR fluorescence properties and in vitro low cytotoxicity observations, we further examined the biocompatibility and organ metabolism of PEI‐CDs in mice using NIR FL imaging. Mice were injected with 200 µL of 15 mg mL^−1^ PEI‐CDs via the tail vein. The major organs (heart, lung, liver, spleen, and kidneys) were excised at different times (0, 0.5, 1, 1.5, 3, 6, 12, and 24 h) after intravenous injections of the PEI‐CDs aqueous solutions for in vitro NIR FL imaging, as shown in Figure 6b. Based on the changes of the NIR FL signals in the main organs, the liver and kidneys could excrete the PEI‐CDs from the body of mice in 24 h after intravenous injections. The cytotoxicity assay and in vitro organ metabolism demonstrated the low toxicity and excellent biocompatibility of the PEI‐CDs.

We further investigated the in vivo NIR imaging in mice via gavage injection. Over time, the bright position of the mouse intestinal tract gradually moved from the front to the middle and end of the intestinal tract, and the intensity of the NIR fluorescence signal gradually decreased (Figure S10, Supporting Information). After 28 h gavage injection, the NIR FL signal disappeared, indicating that PEI‐CDs were gradually excreted through the digestive system. In the in vivo imaging, the bright NIR fluorescence signal from the intestine of the mouse after 1h gavage injection (Figure 6c) exhibited a high signal‐to‐background ratio (S/N) of up to 18, demonstrating the promising ability of in vivo NIR imaging with deep‐tissue penetration.

**Figure 7:**
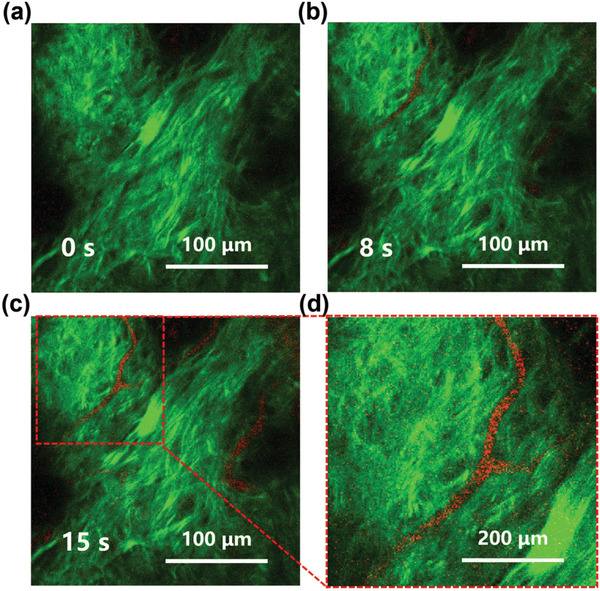
In vivo two‐photon NIR FL angiography in the mouse ear. Two‐photon NIR fluorescence (red color) and second harmonic generation (SHG) images of the blood vessels of the mouse ear a) before and b,c) after (8 and 15 s) intravenous injection of PEI‐CDs aqueous solution (200 µL, 15 mg mL^−1^). d) Regional enlargement of (c).

Considering the significant two‐photon NIR FL properties of PEI‐CDs under the NIR‐II excitation window, in vivo two‐photon NIR FL angiography in the mouse ear was carried out based on PEI‐CDs under 1300 nm fs‐laser excitation. A 200 µL PEI‐CDs aqueous solution (15 mg mL^−1^) was injected into a mouse through the tail vein with an indwelling needle. The second harmonic generation (SHG) signals of collagen fibers (green color in **Figure** **7**) can help identify the region with vessels. The two‐photon NIR emission from the PEI‐CDs appeared in the vessel of the mouse ear after injection (red color in Figure 7). The PEI‐CDs' signals were detectable at 8 s and reached the highest intensity at 15 s after the injection (Figure 7b–d). The above results demonstrate that PEI‐CDs can be used as biocompatible and effective NIR FL probes for in vivo single‐ and two‐photon NIR FL imaging.

## 3 Conclusions

In summary, we for the first time developed a new kind of CDs with both main NIR absorption/emission bands in aqueous solution by fusing large conjugated perylene derivatives with post‐surface engineering. Remarkably, the solvothermal reactions of PTCDA and urea in DMF resulted in CDs with both main NIR absorption bands at 720 nm and NIR emission bands at 745 nm in an aqueous solution. After post‐surface modification with PEI, the absorption and emission peaks of PEI‐CDs in NIR region were redshift and the PLQYs of PEI‐CDs increased to 5.3% and 18.8% in aqueous and DMF solutions, respectively. The PLQY of PEI‐CDs in aqueous media can be further enhanced to 8.3% after combination with BSA, indicating the effective prevention of fluorescence quenching of water. The fusion of large conjugated perylene derivatives to achieve NIR emissive CDs with expanded *π*‐conjugation was further confirmed by DFT calculations. Notably, PEI‐CDs exhibit low cytotoxicity, good biocompatibility, rapid renal clearance, prominent photostability, and much lower cost compared with commercial NIR dye ICG. The remarkable two‐photon NIR angiography in mouse ears and high contrast in vivo NIR fluorescence bioimaging of mice gut demonstrate the potential to be the ideal NIR probes. We expect that the proposed strategy of fusing large conjugated molecules will be a universal method for the development of CDs to promote their bioimaging applications.

## 4 Experimental Section

All chemicals and organic solvents were obtained from commercial suppliers and were used without further purification. Ultrapure water was used in all experiments. The solvothermal reactions were conducted in a Wattcas autoclave (WP‐MSAR‐250A, Figure S11, Supporting Information) or a constant temperature oven. All animal experiments were approved by the University of Macau Animal Ethics Committee (protocol no. UMARE‐015‐2019).

### Synthesis of CDs

0.2g PTCDA and 1g urea were generated in a 50 mL Teflon autoclave with 20 mL DMF before it was put into a 160 ℃ constant temperature oven for 5 h. After the reaction solution was centrifuged at 8000 rpm for 10 min, the obtained upper layer of the dark brown DMF solution was diluted with 3 times ultra‐pure water before purifying with a dialysis bag of 1000 Da for 24 h to remove the residual organic molecules. During the first 3 h of dialysis, the ultrapure water outside the dialysis bag was changed every half hour. After dialysis, the aqueous solution in the dialysis bag was concentrated with a rotary evaporator and freeze‐dried to obtain dark solid CDs (147 mg). The product yield of CDs was 12.3% (w/w).

### Synthesis of PEI‐CDs

0.5g of PEI (1800 MW) was dissolved in the supernatant solution after centrifugation of the synthesized CDs solution and then reacted for another 5 h at 160 ℃. The reaction solution was diluted with 3 times ultra‐pure water and dialyzed for 24 h in a dialysis bag of 20 000 Da. During the first 3 h of dialysis, the ultrapure water outside the dialysis bag was changed every half hour. The dialyzed aqueous solution was concentrated using a rotary evaporator and freeze‐dried to obtain black solid PEI‐CDs (304 mg). The product yield of PEI‐CDs was 17.9% (w/w).

### Characterization

TEM (FEI Tecnai−G2−F30 operated at 200 kV) was used for morphological studies. The UV–vis absorption spectra were collected on a UV–vis–NIR spectrophotometer (Jasco V‐770), and the PL spectra, PL quantum yield, and PL decay of all nanomaterials were collected at room temperature on an Edinburgh FS5 spectrophotometer. The PL lifetime were re‐confirmed on a time‐resolved confocal microscopy (PicoQuant Microtime 200). The PLQYs were measured by an absolute method on an Edinburgh FS5 spectrophotometer equipped with an integrating sphere (SC‐30 module). X‐ray photoelectron spectroscopy measurements were performed using an electron analyzer (ESCALAB 250Xi, Thermo Fisher Scientific) with a UV source (He I*α*, *hυ* = 21.2 eV) for UPS and a monochromatic X‐ray source (Al K*α*, h*υ* = 1486.7 eV) for XPS under ultrahigh‐vacuum conditions (base pressure, 1.0 × 10^−9^ mbar). The fundamental‐frequency 800 nm femtosecond laser pulse was output from the Coherent Legend regenerative amplifier (100 fs, 1 kHz) seeded by a Coherent Vitesse oscillator (100 fs, 80 MHz). The 1300 nm femtosecond laser used in the two‐photon FL and the 600 nm femtosecond laser used in the TA measurement were generated through an automated optical parametric amplifier (Light Conversion, TOPAS Prime) pumped by a fundamental‐frequency 800 nm femtosecond laser pulse. Two‐photon up‐conversion FL signals were focused on a spectrometer (ANDOR, Shamrock 303i) coupled with a CCD (ANDOR, Newton DU920P) through an optical fiber. TA measurements were performed using an Ultrafast System HELIOS spectrometer with a nondegenerate pump‐probe configuration.

### DFT Calculations

The B3LYP density functional method with D3(BJ) dispersion correction was employed in this work to carry out all computations. The 6‐31G(d) basis set was used for the atoms in the geometry optimization. Vibrational frequency analyses at the same level of theory were performed on all the optimized structures to characterize the stationary points as local minima. The Gaussian 16 suite of programs was used throughout this study.

### In Vitro Cytotoxicity Study

Three types of cancer cells (hct116, qbc939, and b477) were used to evaluate the cytotoxicity of PEI‐CDs in the dark using the Alamar blue assay. The cells were seeded in 96‐well plates at a density of 5000 cells per well and cultured overnight. The medium was then replaced with 100 µL fresh medium containing PEI‐CDs at various concentrations (0–16 mg mL^−1^). After incubation for 24 h, the medium was replaced with 100 µL fresh medium containing 10% Alamar blue, and an additional 3–4 h incubation was conducted for the cells. The cell viability was calculated according to the absorbance of the correlated cells at Ex 560 nm and Em 590 nm using a microplate spectrophotometer with the cells only cultured with the medium as the control. Each trial was performed with three wells in parallel.

### In Vivo Metabolism Study

6–8‐week‐old FVB mice were used for the in vivo organ metabolism imaging of the PEI‐CDs aqueous solutions (15 mg mL^−1^) injected intravenously (tail vein) at different time points before the mice were sacrificed to simultaneously obtain organ imaging. The ChemDocTM MP Imaging System (Bio‐Rad Laboratories, Inc.) was used to acquire the images with 680 nm excitation light and a 715/30 nm emission filter. None of the animals exhibited any signs of acute toxicological responses during the experiments.

### In Vivo NIR Fluorescent Imaging

6–8‐week‐old nude mice were used for the NIR fluorescence imaging. The mouse was intragastric with 0.4 mL PEI‐CDs@BSA aqueous solution (PEI‐CDs 15 mg mL^−1^, BSA 15 mg mL^−1^) through gavage injection. An ANDOR iXon Life 888 electron‐multiplying CCD camera coupled with a 750 nm long‐pass optical filter was employed to perform real‐time in vivo NIR fluorescence imaging under an excitation laser of 690 nm. Imaging was performed at various time points.

### Two‐Photon Angiography of PEI‐CDs

6–8‐week‐old mouse was anesthetized by intraperitoneal injection of avertin (0.25 mg g^−1^) before the tail‐vein injection of 200 µL PEI‐CD aqueous solution (15 mg mL^−1^). The mouse was placed on the stage with one ear attached to the bottom of the coverslip, and the glycerol was used as an interstitial medium to reduce optical reflection. A time‐course two‐photon fluorescence and SHG imaging of the mouse ear under 1300 nm laser excitation was recorded in vivo (Ti‐2E A1R‐MP+, Nikon instruments Inc). The femtosecond laser beam was focused onto the ear using a 40× NA = 1.15 water‐immersion objective. The laser power applied to the sample was ≈4.5 mW.

## Conflict of Interest

The authors declare no conflict of interest.

## Supporting information

Supporting Information

## Data Availability

The data that support the findings of this study are available from the corresponding author upon reasonable request.
