# Supplementary material for: Toward Strong Near‐Infrared Absorption/Emission from Carbon Dots in Aqueous Media through Solvothermal Fusion of Large Conjugated Perylene Derivatives with Post‐Surface Engineering
Source: Adv Sci (Weinh). 2022 Jun 2;9(23):2202283. doi: 10.1002/advs.202202283 (PMC9376813; doi:10.1002/advs.202202283)
Supplement: Supplementary file 1 — Supporting Information [file ADVS-9-2202283-s001.pdf]

ADVANCED SCIENCE
Open Access

Supporting Information

for *Adv. Sci.*, DOI 10.1002/advs.202202283

Toward Strong Near-Infrared Absorption/Emission from Carbon Dots in Aqueous Media through Solvothermal Fusion of Large Conjugated Perylene Derivatives with Post-Surface Engineering

*Yupeng Liu, Josh Haipeng Lei, Gang Wang, Zhiming Zhang, Jun Wu, Bohan Zhang, Huiqi Zhang, Enshan Liu, Liming Wang, Tzu-Ming Liu, Guichuan Xing, Defang Ouyang, Chu-Xia Deng, Zikang Tang and Songnan Qu**

Supporting Information for

Toward Strong Near-infrared Absorption/Emission from Carbon Dots in Aqueous Media through Solvothermal Fusion of Large Conjugated Perylene Derivatives with Post-Surface Engineering

*Yupeng Liu[#], Josh Haipeng Lei[#], Gang Wang[#], Zhiming Zhang, Jun Wu, Bohan Zhang, Huiqi Zhang, Enshan Liu, Liming Wang, Tzu-Ming Liu, Guichuan Xing, Defang Ouyang, Chu-Xia Deng, Zikang Tang, Songnan Qu**

Y. Liu, G. Wang, J. Wu, B. Zhang, H. Zhang, E. Liu, Prof. G. Xing, Prof. Z. Tang, Prof. S. Qu

Joint Key Laboratory of the Ministry of Education, Institute of Applied Physics and Materials Engineering, University of Macau, Taipa, Macau SAR, 999078, China.

Dr. J. Lei, Z. Zhang, Prof. T. Liu, Prof. C. Deng

Cancer Center, Faculty of Health Sciences, University of Macau, Macau SAR, 999078, China.

Dr. J. Lei, Z. Zhang, Prof. T. Liu, Prof. C. Deng, Prof. Z. Tang, Prof. S. Qu

MOE Frontier Science Centre for Precision Oncology, University of Macau, Taipa, Macau SAR, 999078, China.

Dr. D. Ouyang

State Key Laboratory of Quality Research in Chinese Medicine and Institute of Chinese Medical Sciences, University of Macau, Taipa, Macau SAR, 999078, China.

Correspondence: songnanqu@um.edu.mo

[#]These authors contributed equally.

Table of contents

Figure S1. FT-IR spectrum of PEI-CDs. **S2**

Figure S2. High-resolution (a) C 1s and (b) O 1s XPS spectra of PTCDA. **S2**

Figure S3. XPS spectra of PEI-CDs. **S3**

Figure S4. UV-vis absorption spectrum of PEI-CDs@BSA in H_2O. **S4**

Figure S5. UV-vis absorption spectrum of different reaction conditions. **S4**

Figure S6. Optical properties of CDs and PEI-CDs. **S5**

Figure S7. Photostability of PEI-CDs compared with Indocyanine green (ICG). **S6**

Figure S8. Two-photon properties of PEI-CDs. **S6**

Figure S9. The PL intensity of PEI-CDs in response to some biological parameters. **S7**

Figure S10. Metabolism of PEI-CDs in mice after intragastric administration. **S7**

Figure S11. The Wattcas autoclave (WP-MSAR-250A) for solvothermal reactions. **S8**

Table S1. Comparison of optical properties of carbon dots with near-infrared absorption and/or emission in aqueous media. **S9**

Table S2. Price comparison of PEI-CDs and commercial NIR dye ICG. **S10**

References. **S11**

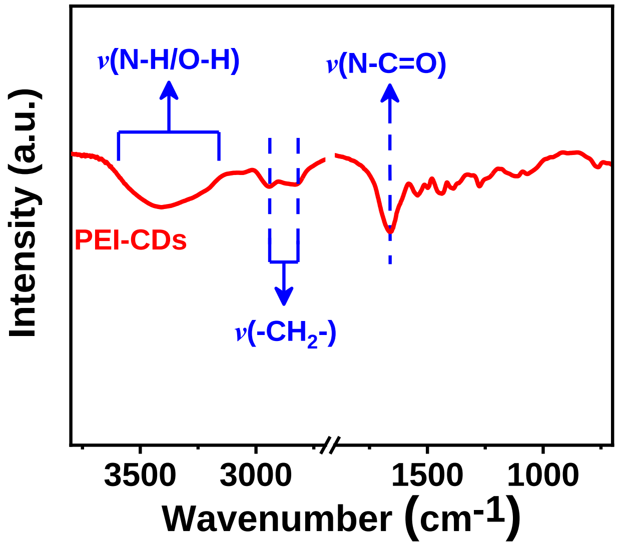

Figure S1. FT-IR spectrum of PEI-CDs.

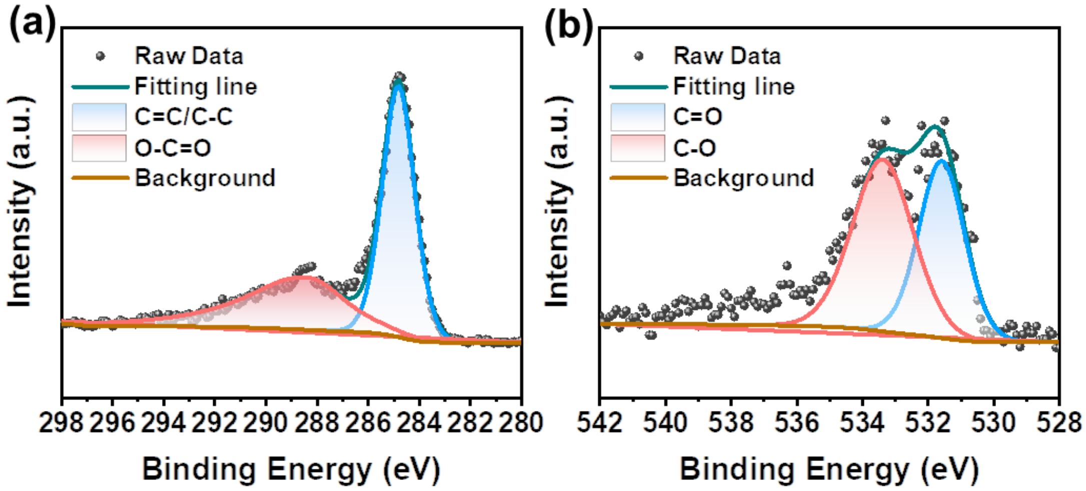

Figure S2. High-resolution (a) C 1s and (b) O 1s XPS spectra of PTCDA.

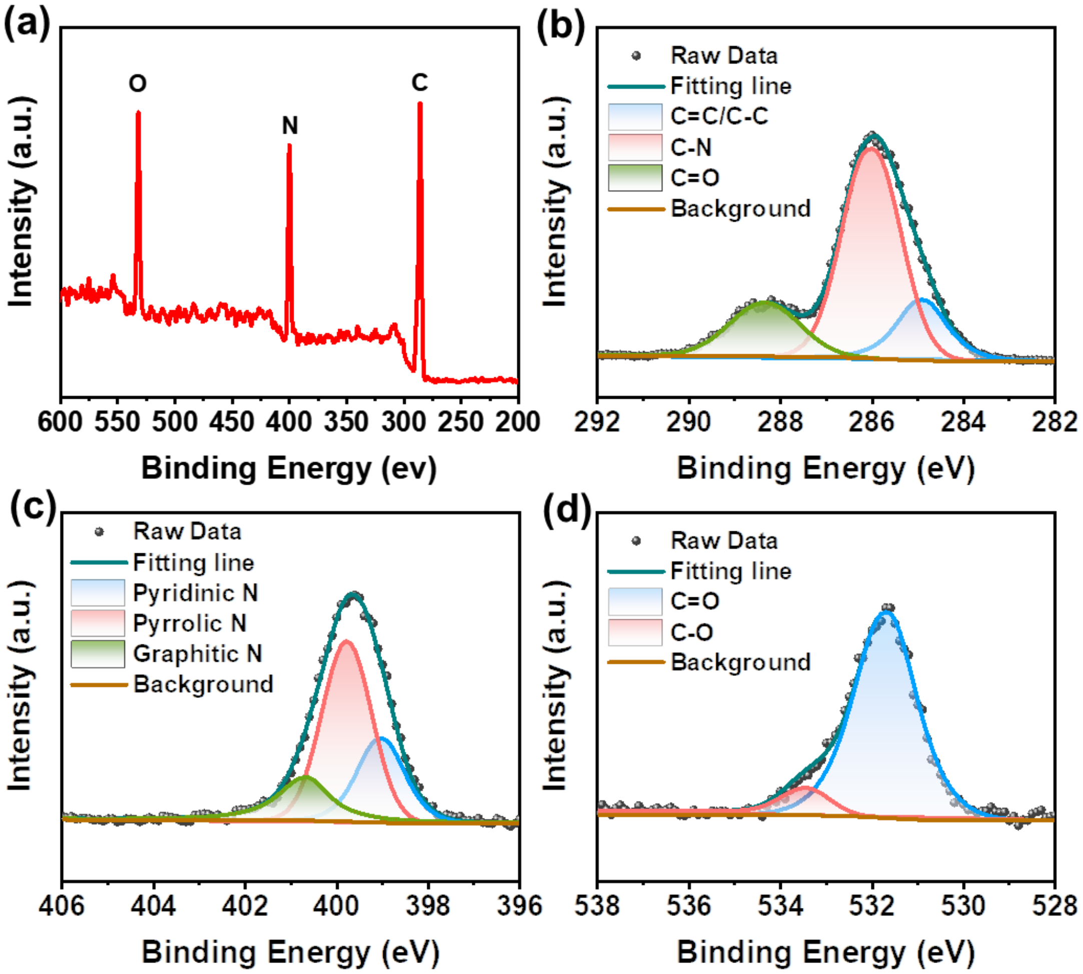

Figure S3. (a) X-ray photoelectron spectroscopy (XPS) survey spectra, (b) high-resolution C 1s XPS spectrum, (c) high-resolution O 1s XPS spectra and (d) high-resolution N 1s XPS spectra of PEI-CDs.

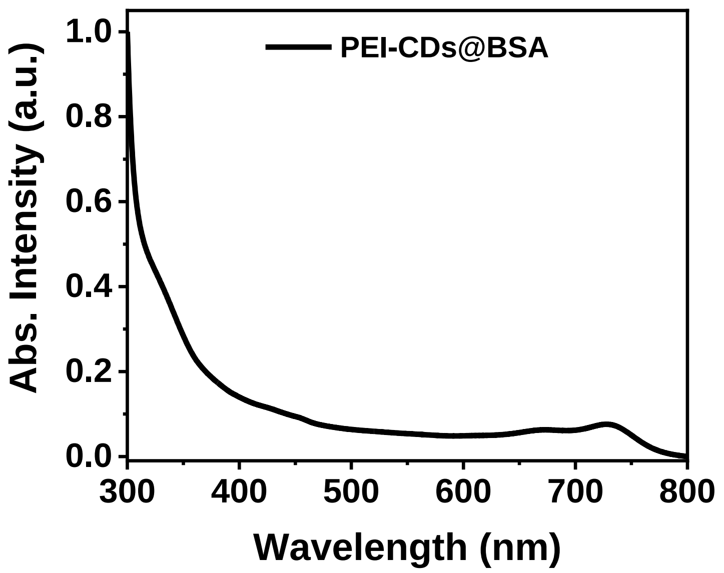

Figure S4. UV-vis absorption spectrum of PEI-CDs@BSA in H_2O.

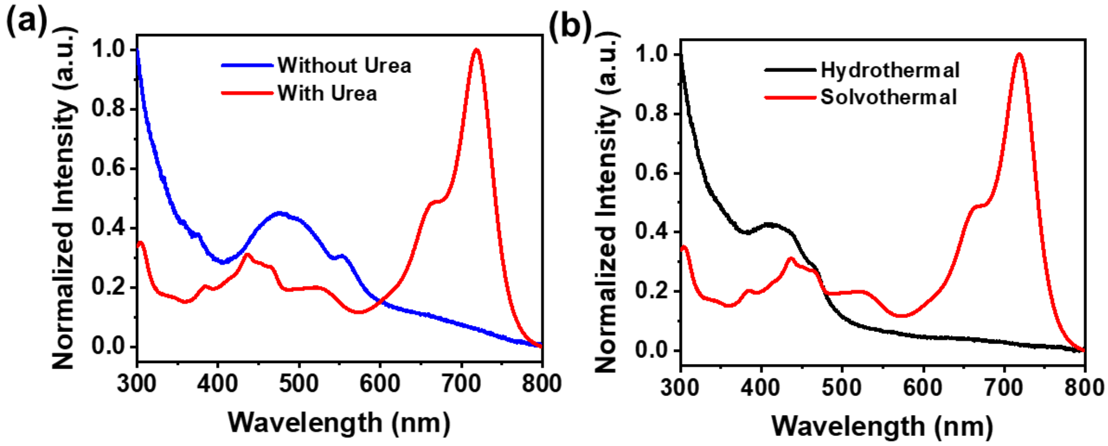

Figure S5. UV-vis absorption spectrum of (a) the comparison of with/without Urea as precursor under solvothermal conditions and (b) the comparison of the reaction between PTCDA and urea under hydrothermal and solvothermal methods.

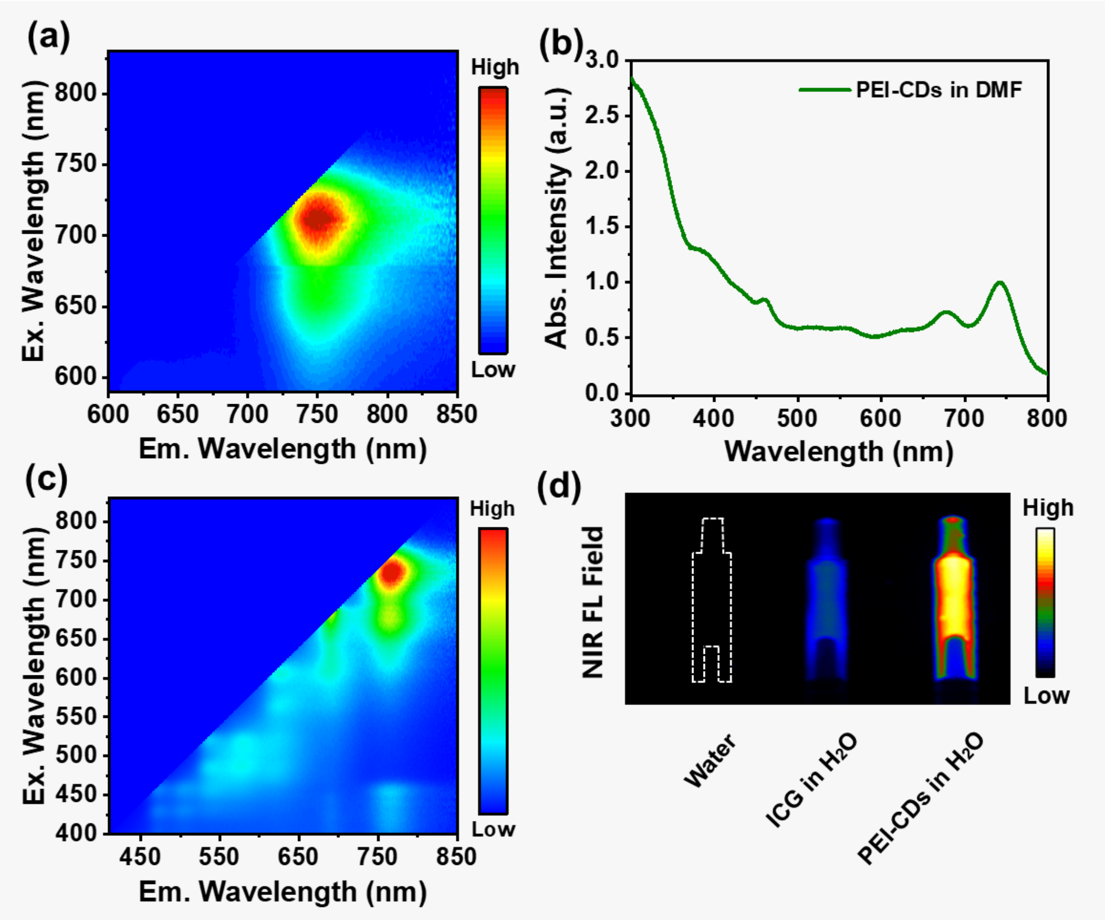

Figure S6. Optical properties of CDs and PEI-CDs. (a) Excitation and emission mapping of CDs in H_2O.UV-vis absorption spectrum (b) and excitation and emission mapping (c) of PEI-CDs in DMF. (d) NIR fluorescence images of water, ICG in H2O, PEI-CDs in H_2O (from left to right) under 690 nm excitation and the emission were recorded with a 750 nm long-pass optical filter.

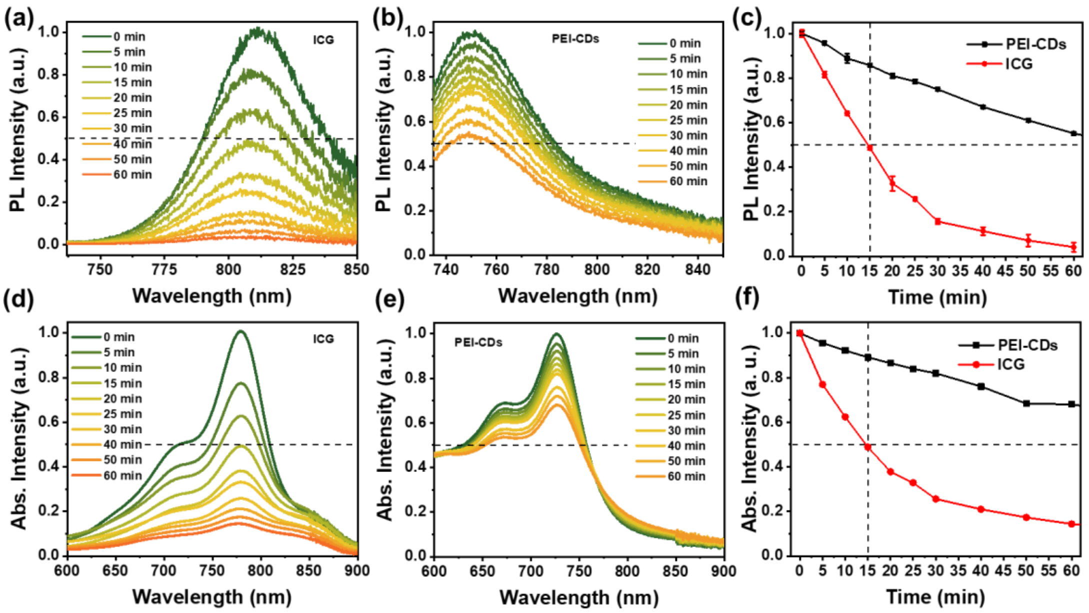

Figure S7. Photostability of PEI-CDs compared with ICG. PL spectra of (a) ICG and (b) PEI-CDs under different radiation times. (c) PL decays of PEI-CDs and ICG under different radiation times. UV-Vis-NIR absorption spectra of (d) ICG and (e) PEI-CDs under different radiation times. (e) Abs. decays of PEI-CDs and ICG under different radiation times. The power density of the laser is 30 mW cm^{-2}.

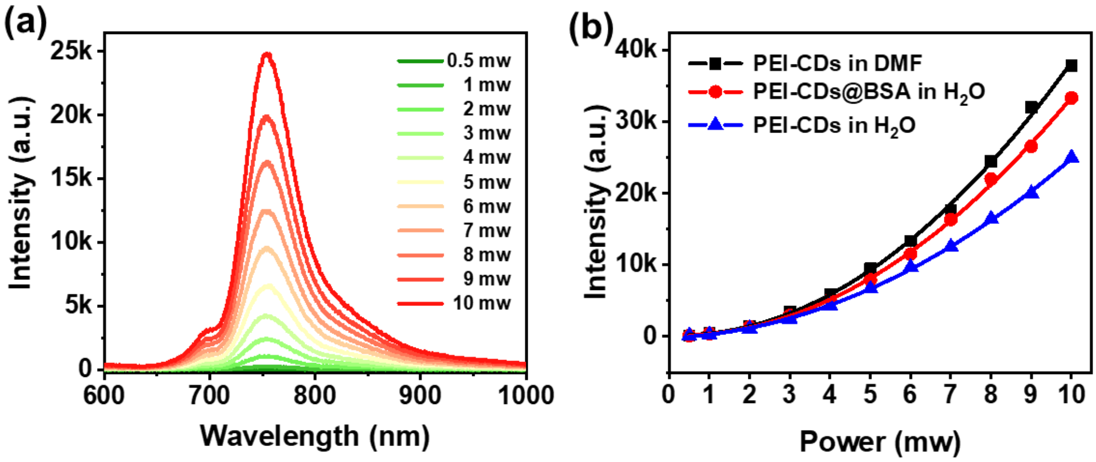

Figure S8. Two-photon properties of PEI-CDs. (a) Two-photon spectrum of PEI-CDs in the aqueous solution (50 mg^{-1}) under the excitation of 1300 nm fs laser with different power. (b) Maximum two-photon FL intensities under different power excitation of PEI-CDs in DMF, PEI-CDs, and PEI-CDs in aqueous solution (PEI-CDs 50 mg^{-1}, BSA 50 mg^{-1}).

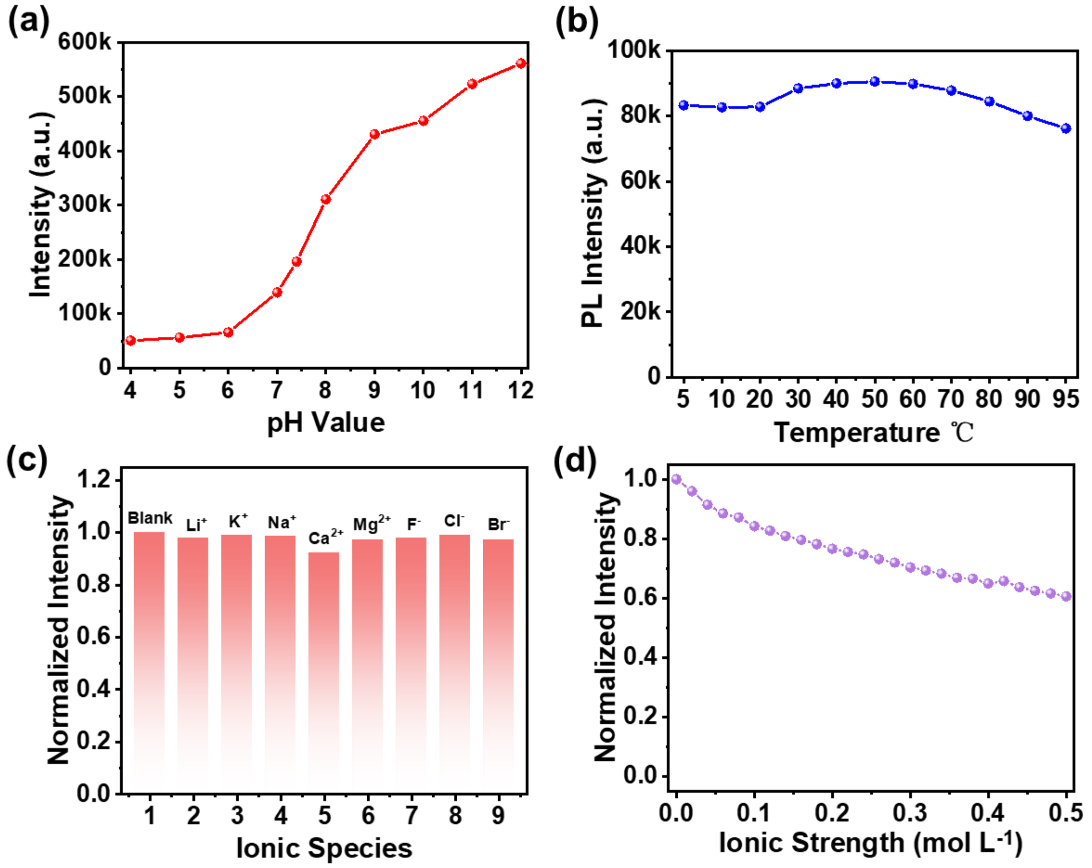

Figure S9. The optical performance of PEI-CDs in response to some biological parameters, (a) different pH values, (b) different temperature, (c) different ionic species, and (d) different ionic strength.

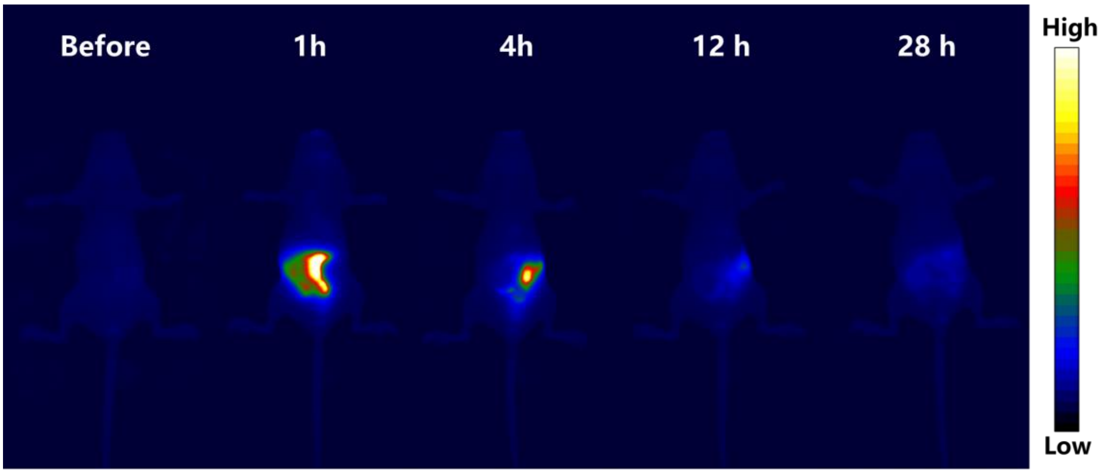

Figure S10. In vivo NIR fluorescence images of a mouse before and after different time gavage injection of PEI-CDs aqueous solution (400 μL, 15 mg mL^{-1}). The imagings were recorded with a 750 nm long-pass optical filter under the excitation of 690 nm.

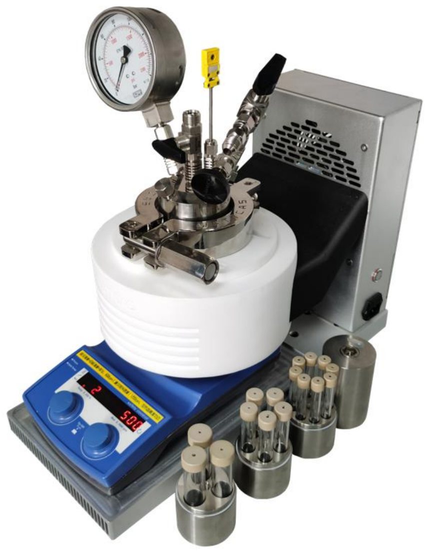

Figure S11. The Wattcas autoclave (WP-MSAR-250A) for solvothermal reactions.

Ref.	Main Abs. peak / nm	Main PL peak / nm	NIR λem / nm (λex / nm)	NIR PLQY in aqueous media	Published Journal Year
**This Work**	720	745	745 (λex 720)	3.3% (CDs)<br>5.3% (PEI-CDs)<br>8.3% (PEI-CDs@BSA)	-
**1**	UV region	440	925 (λex 808 laser)	0.4%	ACS Appl. Mater. Interfaces 2019
**2**	UV region	470	1000 (λex 808 laser)	~1%	Appl. Mater. Today 2019
**3**	UV region	532	950 (λex 808 laser)	1.34%	2D Mater. 2021
**4**	UV region	532	728 (λex 300)	N/A[a]	ACS Appl. Mater. Interfaces 2018
**5**	UV region	410	915 (λex 808 laser)	N/A	ACS Nano 2014
**6**	UV region	475	N/A	N/A	Carbon 2018
**7**	UV region	470	N/A	N/A	Carbon 2020

[a] N/A: not available.

Table S1. Comparison of optical properties of carbon dots with near-infrared absorption/emission in aqueous media.

	Cost	Total	Ref.
**ICG**	¥25,160 / 1g	¥25,160 / 1g	8<br>(package 250mg)

	Cost	Total	
Urea	¥0.71 / 1g		9<br>(package 500g)
PTCDA	¥1.93 / 0.2g		10<br>(package 100g)
PEI	¥4.84 / 1mL (50% w/w)		11<br>(package 250mL)
DMF	¥28.12 / 20mL		12<br>(package 1L)
**PEI-CDs**	¥35.60 / 0.3 g	¥118.67 / 1g	

Table S2. Price comparison of PEI-CDs and commercial NIR dye Indocyanine green (ICG).

References

[1] Theranostic Carbon Dots with Innovative NIR-II Emission for in Vivo Renal-Excreted Optical Imaging and Photothermal Therapy, **ACS Appl. Mater. Interfaces,** 2019, *11*, 4737.

[2] Nitrogen and boron dual-doped graphene quantum dots for near-infrared second window imaging and photothermal therapy, **Applied Materials Today,** 2019, *14*, 108.

[3] Near-infrared emitting graphene quantum dots synthesized from reduced graphene oxide for in vitro/in vivo/ex vivo bioimaging applications, **2D Mater.,** 2021, *8*, 035013.

[4] Near-Ultraviolet to Near-Infrared Fluorescent Nitrogen-Doped Carbon Dots with Two-Photon and Piezochromic Luminescence, **ACS Appl. Mater. Interfaces,** 2018, *10*, 33, 27920.

[5] Deep Ultraviolet to Near-Infrared Emission and Photoresponse in Layered N-Doped Graphene Quantum Dots, **ACS Nano,** 2014, *8*, 6312.

[6] NIR-responsive carbon dots for efficient photothermal cancer therapy at low power densities, **Carbon,** 2018, *134*, 153.

[7] Enriched graphitic N dopants of carbon dots as F cores mediate photothermal conversion in the NIR-II window with high efficiency, **Carbon,** 2020, *162*, 220.

[8] https://www.sigmaaldrich.cn/CN/en/product/usp/1340009

[9] https://www.sigmaaldrich.cn/CN/en/product/sial/u1250

[10] https://www.sigmaaldrich.cn/CN/en/product/aldrich/p11255

[11] https://www.sigmaaldrich.cn/CN/en/product/aldrich/408700

[12] https://www.sigmaaldrich.cn/CN/en/product/sial/227056
